# Housing conditions of urban households with Aboriginal children in NSW Australia: tenure type matters

**DOI:** 10.1186/s12889-017-4607-y

**Published:** 2017-08-01

**Authors:** Melanie J Andersen, Anna B Williamson, Peter Fernando, Darryl Wright, Sally Redman

**Affiliations:** 1 School of Public Health and Community Medicine, The University of New South Wales, Sydney, 2052 Australia; 2 The Sax Institute, 235 Jones St, Haymarket, Sydney, 2007 Australia; 3 Tharawal Aboriginal Corporation, 187 Riverside Dr, Airds, 2560 Australia

**Keywords:** Aboriginal, Indigenous, Housing, Urban, Environmental health, Tenure, Disadvantage

## Abstract

**Background:**

Housing is a key determinant of the poor health of Aboriginal Australians. Most Aboriginal people live in cities and large towns, yet research into housing conditions has largely focused on those living in remote areas. This paper measures the prevalence of housing problems amongst participants in a study of urban Aboriginal families in New South Wales, Australia, and examines the relationship between tenure type and exposure to housing problems.

**Methods:**

Cross-sectional survey data was provided by 600 caregivers of 1406 Aboriginal children aged 0–17 years participating in Phase One of the Study of Environment on Aboriginal Resilience and Child Health (SEARCH). Regression modelling of the associations between tenure type (own/mortgage, private rental or social housing) and housing problems was conducted, adjusting for sociodemographic factors.

**Results:**

The majority (60%) of SEARCH households lived in social housing, 21% rented privately and 19% either owned their home outright or were paying a mortgage (“owned”). Housing problems were common, particularly structural problems, damp and mildew, vermin, crowding and unaffordability. Physical dwelling problems were most prevalent for those living in social housing, who were more likely to report three or more physical dwelling problems than those in owned (PR 3.19, 95%CI 1.97, 5.73) or privately rented homes (PR 1.49, 1.11, 2.08). However, those in social housing were the least likely to report affordability problems. Those in private rental moved home most frequently; children in private rental were more than three times as likely to have lived in four or more homes since birth than those in owned homes (PR 3.19, 95%CI 1.97, 5.73). Those in social housing were almost half as likely as those in private rental to have lived in four or more homes since birth (PR 0.56, 95%CI 0.14, 0.77). Crowding did not vary significantly by tenure type.

**Conclusions:**

The high prevalence of housing problems amongst study participants suggests that urban Aboriginal housing requires further attention as part of efforts to reduce the social and health disadvantage experienced by Aboriginal Australians. Particular attention should be directed to the needs of those renting in the private and social housing sectors, who are experiencing the poorest dwelling conditions.

**Electronic supplementary material:**

The online version of this article (doi:10.1186/s12889-017-4607-y) contains supplementary material, which is available to authorized users.

## Background

There is a large body of international evidence that housing environments affect human health, productivity and wellbeing [1–6]. Housing disadvantage can take many forms, including poor physical dwelling conditions, crowding, instability and unaffordability [3, 7]; thus, it can affect people through myriad direct and indirect pathways [8]. For instance, dampness is associated with respiratory illness, crowding with stress and infectious disease, frequent residential moves with poor child education outcomes and unaffordability with poor mental health [6, 7, 9]. Multiple forms of housing problems can also coexist and have a compounded, cumulative impact on physical and mental health, particularly after prolonged exposure during childhood [1, 7, 10, 11].

Aboriginal Australians experience significant disadvantage on many social indicators compared to non-Aboriginal Australians, including health, life expectancy, education, employment, imprisonment and housing [12]. Inadequate housing has long been considered a key determinant of the poor health status of Aboriginal and Torres Strait Islander Australians [13–15] (hereafter Aboriginal). Similar relationships between poor housing and poor health have been noted for first nations peoples in other wealthy countries with histories of colonisation and dispossession, such as Canada, New Zealand and the United States of America [16–19]. Many Aboriginal communities in remote parts of Australia experience high rates of severe overcrowding, homelessness and very poor dwelling conditions. In many remote communities, basic amenities required to engage in ‘Healthy Living Practices’ (HLPs), including flushing toilets, facilities required to bathe, wash clothes or prepare and store food adequately, have been found missing or non-functional in a substantial proportion of households [15, 20–25].

While the housing problems experienced by many Aboriginal people in remote communities are of obvious and pressing concern, the majority (79%) of Aboriginal Australians live in major cities and large regional centres [26]. Yet few studies have specifically examined the housing conditions experienced by urban Aboriginal people. This parallels a broader dearth of research about the health of urban Aboriginal Australians, even though their burden of illness is higher than that of remote Aboriginal people [27–29]. Moreover, Baker et al. cite a more widespread under-acknowledgement of housing problems in the general Australian population [3]. Recent research found that the ‘hidden fraction’ of Australians living in poor quality housing is actually quite substantial; over 100,000 Australians are estimated to live in housing classified as ‘very poor-derelict’ and Aboriginal Australians were three times more likely than non-Aboriginal Australians to live in dwellings meeting this classification [3].

The existing body of research into urban Aboriginal housing conditions is scant, but what is known suggests the need for further attention. Qualitative research highlights housing as an issue of significant concern for urban Aboriginal Australians [30–33]. Urban Aboriginal people fare notably worse on most available housing indicators than their non-Aboriginal neighbours in national surveys, with higher rates of household crowding, homelessness and need for repairs [34, 35]. Aboriginal-specific surveys provide greater detail about dwelling conditions and reveal that the proportion of urban Aboriginal households experiencing crowding and poor dwelling conditions is lower than for those in remote communities [35–37]. However, in absolute terms, higher numbers of those Aboriginal people experiencing overcrowding live in cities and towns and some housing issues such as unaffordability, homelessness and frequent residential moves are more prevalent for urban Aboriginal people [36, 38]. Over 8% of the Perth metropolitan homes surveyed in the Western Australian Aboriginal Child Health Survey (WAACHS) were classified as being in poor condition, having failed three or more of the eight indicators of basic household functioning required to perform HLPs [36].

Tenure type describes the nature of the legal right a householder has to occupy the dwelling in which they live [39]. In Australia, private home ownership is the dominant tenure type. At the 2011 census, 68% of non-Aboriginal households owned their home, either outright (33%) or with a mortgage (35%), and less than a third (29%) were renting in some capacity [35]. Aboriginal households are much less likely to own their home outright (11%) or be paying off a mortgage (25%) and are instead much more likely to be renting (59%). Aboriginal households are also six times as likely to be renting their home from a social housing provider as other Australian households (26% vs 4% respectively) [35]. That said, home ownership rates are higher for Aboriginal people in urban than remote areas, with rates slowly rising amongst a relatively small but growing number of professional and middle-income Aboriginal households [40, 41].

As noted by Bentley et al., tenure type has particular significance in Australia where,

*“there is a long history of a preference for home ownership, while private rental is widely regarded as a tenure of transition towards homeownership and social housing is solidly seen as a welfare ‘safety net’ for those unable to own or rent in the private market.”* p5 [42].

Home ownership in Australia brings many social and financial benefits but is not an option available to everyone [38]. In qualitative research with Aboriginal residents of Western Sydney, participants described social housing as the only feasible tenure type for many urban Aboriginal people, as neither home ownership nor private rental were financially accessible and additional barriers such as racial discrimination were experienced in the private rental market [31]. While social housing was generally described as a desirable tenure type for the relative stability and affordability it offered, participants living in social housing reported poor dwelling conditions [31]. These reports accord with existing evidence which suggests that tenure type and landlord type are associated with significant differences in housing conditions in both Aboriginal and general populations [3, 37, 43, 44].

The limitations of existing urban Aboriginal housing data sources include the under-identification of Aboriginal people in population surveys [35, 45], small urban sub-populations in the two main Aboriginal child health studies that include housing questions [36, 46] and varying levels of detail about housing conditions [47]. For instance, in one of these studies, only three housing variables were used: carer response to, ‘in the last year have you felt too crowded where you live, moved house or had housing problems’ (y/n); ‘overcrowding’, (more than two persons per bedroom); ‘home needs major repairs’ (y/n) [47]. These measures were described as insufficiently precise [47]. More detailed information about the type and number of housing problems can be important when examining the interactions between housing and health [11, 25, 47]. Understanding the prevalence and distribution of the particular housing problems facing urban Aboriginal people is vital for informing housing and health policy aiming that aims to close the gap on Aboriginal disadvantage.

This paper aims to quantitatively describe the housing situations of families participating in the Study of Environment on Aboriginal Resilience and Child Health (SEARCH), the largest urban Aboriginal child health cohort study ever conducted in Australia. Phase one SEARCH data is used to examine cross-sectional associations, firstly between sociodemographic factors and tenure type, then between tenure type and specific housing problems, adjusting for relevant sociodemographic factors.

## Methods

### Sampling strategy

SEARCH is investigating the causes of health and illness in urban Aboriginal children, with a particular focus on the health priorities identified by participating Aboriginal communities: infectious disease; otitis media; mental health; injury; developmental delay; obesity; and risk factors for chronic disease [48]. SEARCH is being conducted in partnership with four Aboriginal Community Controlled Health Services (ACCHS) located in urban and large regional centres in the state of New South Wales (NSW): Mount Druitt (Aboriginal Medical Service Western Sydney); Campbelltown (Tharawal Aboriginal Corporation); Wagga Wagga (Riverina Medical and Dental Aboriginal Corporation), and Newcastle (Awabakal Ltd.) [48].

Aboriginal children aged 0–17 years and their parents or caregivers were recruited through their local Aboriginal Community Controlled Health Services (ACCHS) between 2008 and 2010. All attending Aboriginal children were eligible to participate in the Phase One survey if their parent or caregiver (hereafter carer) was aged 16 years or over and was willing to provide contact information for follow up interviews. Carers of eligible Aboriginal children did not have to be Aboriginal. Families were invited to participate in SEARCH when presenting at a participating ACCHSs by an Aboriginal research officer, or informed about the study by their doctor or health worker [48]. No response rate data was kept. The SEARCH cohort will be followed for twenty years, funding permitting, with data collected on all outcomes and exposures - including housing – every five years, as outlined in the study protocol [48]. The sample for the current study includes all carers who completed a survey about their housing during Phase One of SEARCH.

### Ethical approvals

SEARCH is a partnership between researchers, ACCHSs and the Aboriginal Health and Medical Research Council (AH&MRC) the peak body for ACCHSs in NSW. The study was approved by the ethics committees of the Aboriginal Health and Medical Research Council of New South Wales (reference 586/06) and of the University of Sydney (reference, 12–2003/9429).

### Measures

SEARCH carers completed a comprehensive survey about their children’s physical and mental health, development, nutritional intake and exercise habits. Carers also completed a questionnaire about themselves covering a range of demographic, social, lifestyle and environmental factors, including their housing [48] (see Additional files 1 and 2). Housing questions were drawn from several established sources, including the NATSISS, WAACHS, Australian Housing Survey and New South Wales Child Health Survey, or developed specifically for SEARCH in consultation with Aboriginal community leaders. No existing self-report housing questionnaires were considered appropriate for unaltered use in an urban Aboriginal context.

### Tenure type

Three main tenure type categories are examined in these analyses: own/mortgage (or “owned”), private rental and social housing (See Additional file 2). Owned homes include those either owned outright or with a mortgage currently being paid by the carer or any usual member of the household. Private rental homes are rented through a real estate or other private tenancy agreement. Social housing homes are those rented from any social housing provider, including mainstream public housing, state owned and managed Indigenous housing or any community housing provider, Aboriginal or non-Aboriginal.

### Other Housing Variables

Survey questions about housing mobility, household size, crowding, affordability, dwelling type and dwelling quality have been categorised for analysis as per Additional file 2. Housing mobility is conceptualised in various ways in the literature [49]. In this study, housing mobility relates to moves between dwellings but not necessarily geographical area. Crowding is a complex construct to quantify in cross-cultural contexts [50] and is measured disparately in existing Aboriginal health studies [25, 36, 47]. SEARCH quantifies several dimensions of household size, occupancy level and crowding, including carer subjective report of feeling crowded and the objective measure persons per bedroom, used as a continuous ratio and binary measure. Homes with more than two persons per bedroom (PPB) are considered crowded as this violates the first condition of the Canadian National Occupancy Standard [47]). The presence of specific physical dwelling problems was reported by carers within eight key domains: structural problems, major electrical problems, major plumbing problems, damp and mildew, poor physical security, vermin, inadequate temperature control and the absence of functioning smoke alarm. A tally of the total number of physical dwelling problem domains was created and used as both a numeric score and binary outcome variable (3+ physical dwelling problems indicating poor physical dwelling conditions) [36].

### Statistical analysis

Sample characteristics are presented as frequencies and percentages or medians and quartiles, as most numeric measures were non-normally distributed. Regression analyses were conducted to examine the relationships between tenure type, housing conditions and socio-economic circumstances. Firstly, multinomial logistic regression models were used to calculate the associations between sociodemographic covariates and tenure type, as this dependant variable had three nominal categories: social housing; private rent; and own/mortgage. The adjusted Odds Ratios of living in each of the three tenure types were calculated for each carer sociodemographic covariate (age, sex, Aboriginality, ACCHS, employment status, qualifications, fortnightly income), adjusting for all sociodemographic covariates to estimate independent associations (Table 1).

**Table 1:** Sociodemographic characteristics of carers who completed the Phase One SEARCH Housing Questionnaire: prevalence and adjusted Odds Ratio by tenure type€

Characteristic	Overall ^a c^	Prevalence by tenure type (%)			aOR (95% CI) ^h^		
		Own/mortgage μ^c^	Private Rent ^bc^	Social Housing ^bc^	Social Housing vs Own/mortgage	Social Housing vs Private rent	Private Rent vs Own/mortgage
Total	600	116 (19%)	125 (21%)	359 (60%)			
Gender							
Male	9.3%	12%	9.6%	8.4%	^e^	^e^	^e^
Female	91%	88%	90%	92%	1.02 (0.44, 2.37)	1.03 (0.45, 2.36)	0.98 (0.39, 2.52)
Age ^d^ ***	33 yrs. (27, 39)	35 yrs. (30, 43)	31 yrs. (26, 37)	33 yrs. (27, 39)	0.70 (0.52,0.93)* ^f^	1.37 (1.04, 1.80)* ^f^	0.51 (0.36,0.71)*** ^f^
Aboriginal & Torres Strait Islander Status ***							
Non-Aboriginal	22%	37%	30%	15%	^e^	^e^	^e^
Aboriginal &/or Torres Strait Islander	78%	64%	70%	86%	4.17 (2.25, 7.74) ***	2.79 (1.60, 4.86)***	1.50 (0.80, 2.80)
Aboriginal Community Controlled Health Service (ACCHS) ***							
ACCHS A	24%	33%	22%	22%	^e^	^e^	^e^
ACCHS B	21%	23%	34%	16%	2.05 (0.94, 4.50)	0.50 (0.25, 0.98)*	4.12 (1.83, 9.30)**
ACCHS C	27%	24%	21%	30%	2.02 (0.97, 4.24)	1.27 (0.64, 2.52)	1.59 (0.70, 3.62)
ACCHS D	28%	20%	24%	32%	4.44 (2.03, 9.73) ***	1.59 (0.80, 3.13)	2.80 (1.18, 6.66)*
Employment Status ***							
Employed	30%	54%	38%	19%	^e^	^e^	^e^
Studying	3.5%	3.4%	4.0%	3.4%	2.22 (0.60, 8.28)	2.38 (0.70, 8.14)	0.93 (0.22, 3.96)
Home duties	54%	34%	48%	63%	3.50 (1.85, 6.65)***	2.11 (1.19, 3.75)*	1.66 (0.84, 3.30)
Not working ^g^	13%	8.6%	11%	15%	3.20 (1.16, 8.83)*	2.18 (0.94, 5.03)	1.47 (0.48, 4.48)
Qualifications *							
Bachelor or Post graduate degree	7.5%	15%	11%	3.9%	^e^	^e^	^e^
Trade, certificate, diploma	41%	63%	47%	31%	0.86 (0.32, 2.33)	1.33 (0.49, 3.66)	0.65 (0.24, 1.74)
None	52%	23%	42%	65%	3.50 (1.21, 10.15)*	2.45 (0.87, 6.91)	1.43 (0.48, 4.28)
Fortnightly Income***							
$2000+	9.8%	26%	11%	4.4%	^e^	^e^	^e^
$800–1999	43%	52%	49%	37%	2.95 (1.27, 6.86)*	1.33 (0.54, 3.29)	2.21 (0.95, 5.17)
$0–799	48%	22%	41%	58%	7.33 (2.95, 18.16)***	1.95 (0.78, 4.89)	3.75 (1.47, 9.59)**

^a^ Percentage of carers overall with each characteristic

^b^ Percentage of carers within each tenure type with each characteristic (column percentages)

^c^ Percentages may not add up to 100 due to rounding

^d^Median (Q1, Q3)

^e^ Referent group

*p < 0.05 **p < 0.01 ***p < 0.001

^f^ Odds of living in tenure type are for each additional 10 years of age ^g^ Not working included carers who reported being unemployed, unable to work or retired

^h^ adjusted for all demographic characteristics: sex, age, ATSI Status, ACCHS, employment status, qualifications & income

Secondly, a series of generalised linear models were used to estimate the adjusted Prevalence Ratios of having each housing characteristics (outcome variable) by tenure type, adjusting for age, sex, ACCHS, Aboriginality and income (Tables 2 and 3). Analyses were conducted firstly with ownership, then private rental as the referent group to specifically examine differences between private rental and social housing, a potentially policy-relevant query. Prevalence Ratios were calculated in favour of Odds Ratios where possible, as they provide a more conservative estimation of effect size where event outcomes are common (>10%) [51], which was the case for most housing variables. In the case of vermin and the categorical ‘number of houses lived in’ variable, binary logistic regression models were instead performed due to issues with convergence. Hence the associations reported for vermin and 4+ homes lived in are adjusted Odds Ratios, as marked in Table 3. Univariate analyses were conducted with all available cases and complete case analysis was conducted with multivariable modelling. All analyses were conducted in SPSS 22.0 [52].

**Table 2:** Housing and household characteristics reported by SEARCH carers and adjusted Prevalence Ratio of each characteristic by tenure type€

		Prevalence by tenure type n (%)			aPR (95% CI) ^g^		
Characteristic	Overall^ac^	Own/mortgage^bc^	Private Rent^bc^	Social Housing^bc^	Social Housing vs Own/mortgage^e^	Social Housing vs Private rent^e^	Private Rent vs Own/mortgage^e^
Total	600	116 (19%)	125 (21%)	359 (60%)			
Dwelling structure * ^b^							
House	94%	99%	89%	94%			
Apartment	6.5%	0.9%	12%	6.5%			
Years in current dwelling*** ^d^	3.0 (1.0, 7.0)	5.0 (1.4, 10.0)	1.1 (0.5, 2.7)	3.7 (1.0, 7.3)	0.84 (0.67, 1.05)	2.28 (1.72, 3.04)***	0.37 (0.27, 0.49)***
Forced to move out in past 12 months	12%	9.5%	14%	13%	0.94 (0.47, 1.89)	0.88 (0.51, 1.55)	1.07 (0.49, 2.34)
Affordability problems ^f^ **	32%	39%	40%	27%	0.65 (0.48, 0.91)**	0.61 (0.46, 0.82)**	1.07 (0.77, 1.49)
Number of people who normally sleep in home ^d^ **							
	5 (4, 6)	5 (4, 6)	4 (3, 6)	5 (4, 6)	1.02 (0.93, 1.12)	1.12 (1.03, 1.23)**	0.91 (0.82, 0.99)*
Number of bedrooms ***							
0–2	6.7%	3.4%	19%	3.4%			
3	59%	45%	54%	65%			
4+	35%	52%	27%	32%			
Median ^d^ ***	3 (3, 4)	4 (3, 4)	3 (3, 4)	3 (3, 4)	0.96 (0.91, 1.01)	1.10 (1.04, 1.16)***	0.87 (0.82, 0.93)***
Persons Per Bedroom (PPB) ^d^	1.3 (1.0, 1.7)	1.3 (1.0, 1.7)	1.5 (1.0, 1.8)	1.3 (1.0, 1.7)	1.02 (0.94, 1.11)	0.97 (0.89, 1.06)	1.05 (0.96, 1.16)
> 2 Persons Per Bedroom (CNOS)^h^	9.0%	6.9%	9.8%	9.4%	0.97 (0.45, 2.37)	0.95 (0.50, 1.92)	1.03 (0.43, 2.60)
Felt too crowded where you live	30%	21%	30%	33%	1.26 (0.93, 1.89)	1.07 (0.78, 1.50)	1.19 (0.76, 1.92)
Home too small *	42%	35%	35%	46%	1.33 (1.00, 1.83)	1.39 (1.06, 1.87)*	0.96 (0.67, 1.39)
Home too big	1.7%	0.9%	1.6%	2.0%			
*Children of carers in current study*	*1406*	*244 (17%)*	*276 (20%)*	*886 (63%)*			
*Homes lived in since birth median* ^*d*^ ***	*2 (1, 4)*	*2 (1, 3)*	*3 (2, 5)*	*2 (1, 4)*	1.19 (1.05, 1.35)**	0.82 (0.75, 0.90)***	1.45 (1.27, 1.67)***
*1–3 homes*	*65%*	*74%*	*54%*	*67%*	^*e*^	^*e*^	^*e*^
*4+ homes* ^i^***	*35%*	*26%*	*46%*	*33%*	1.87 (1.25, 2.78)**	0.56 (0.41, 0.77)***	3.31 (2.14, 5.13)***

a Percentage of households with each housing characteristic overall

^b^ Percentage of households within each tenure type with each housing characteristic

^c^ Percentages may not add up to 100 due to rounding

^d^ Median (Q1, Q3)

^e^Referent group

*p < 0.05 **p < 0.01 ***p < 0.001

^f^ Affordability problems if reported yes to rent, rates or mortgage too high

^g^ adjusted for age, sex, ACCHS, Aboriginality and income

^h^ Households considered crowded as per the first Canadian National Occupancy Standard, having more than 2 persons per bedroom

^i^ Adjusted Odds Ratio not Prevalence Ratio

**Table 3:** Physical dwelling problems reported by SEARCH carers and adjusted Prevalence Ratio of each characteristic by tenure type €

		Prevalence by tenure type n (%)			aPR (95% CI) €		
Housing Problem Present (Yes)	Overall^ac^	Own/mortgage^bc^	Private Rent ^bc^	Social Housing^bc^	Social Housing vs Own/mortgage^e^	Social Housing vs Private rent^e^	Private Rent vs Own/mortgage^e^
	600	116 (19%)	125 (21%)	359 (60%)			
Structural problems **	40%	23%	32%	48%	1.73 (1.23, 2.60)**	1.30 (0.99, 1.78)	1.34 (0.88, 2.09)
Sinking/moving foundations *	20%	11%	13%	26%	1.80 (1.06, 3.36)*	1.75 (1.08, 3.07)*	1.03 (0.51, 2.11)
Major cracks in walls/floors *	29%	14%	23%	35%	1.85 (1.17, 3.16)*	1.27 (0.91, 1.87)	1.45 (0.85, 2.60)
Sagging floors *	12%	4.4%	8.3%	16%	3.22 (1.40, 9.37)*	1.69 (0.89, 3.64)	1.90 (0.67, 6.14)
Walls or windows not straight **	17%	7.9%	7.4%	24%	2.66 (1.44, 5.66)**	2.78 (1.52, 5.85)**	0.96 (0.39, 2.38)
Wood rot/termite damage **	17%	4.4%	9.9%	24%	5.72 (2.39, 18.75)**	2.11 (1.22, 4.01)*	2.71 (0.97, 9.53)
Damp and Mildew***	34%	13%	28%	43%	2.90 (1.83, 5.00)***	1.53 (1.12, 2.18)*	1.89 (1.11, 3.41)*
Rising damp ***	19%	6.3%	16%	25%	3.36 (1.68, 7.99)**	1.44 (0.91, 2.41)	2.34 (1.06, 5.87)
Damp/mildew on walls, ceilings, windows***	33%	12%	27%	42%	2.92 (1.81, 5.17)***	1.52 (1.10, 2.19)*	1.92 (1.10, 3.56)*
Major electrical problems	13%	5.3%	8.3%	17%	2.39 (1.11, 6.24)	1.86 (0.98, 4.00)	1.29 (0.48, 3.76)
Major plumbing problems	16%	5.3%	12%	20%	2.30 (1.10, 5.91)	1.20 (0.71, 2.19)	1.92 (0.80, 5.27)
No functioning smoke alarm installed *	6.5%	7.8%	11%	4.3%	0.32 (0.13, 0.83)*	0.37 (0.16, 0.84)*	0.85 (0.37, 2.11)
Needs to be more secure ***	39%	12%	28%	51%	3.30 (2.03, 5.96)***	1.67 (1.24, 2.36)**	1.98 (1.13, 3.72)*
Vermin (cockroaches, mice or other) ^g^***	43%	20%	34%	54%	4.24 (2.39, 7.52)***	2.49 (1.53, 4.06)***	1.70 (0.90, 3.25)
Temperature Control **	27%	9.5%	25%	34%	3.26 (1.78, 6.83)**	1.30 (0.92, 1.92)	2.50 (1.29, 5.43)*
Unable to make home warm enough in winter*	18%	7.8%	16%	22%	2.05 (1.10, 4.33)*	1.26 (0.82, 2.05)	1.63 (0.80, 3.61)
Unable to make home cool enough in summer***	22%	6.9%	18%	28%	3.95 (1.90, 10.10)**	1.46 (0.95, 2.37)	2.71 (1.20, 7.23)*
^h^ No. of physical dwelling problems***^d^	1 (0, 4)	0 (0, 1)	1 (0, 3)	2 (1, 4)	2.29 (1.63, 3.21)***	1.46 (1.18, 1.80)***	1.57 (1.09, 2.27)*
0	31%	56%	34%	22%	^e^	^e^	^e^
1	19%	22%	22%	17%	^e^	^e^	^e^
2	13%	9.5%	15%	13%	^e^	^e^	^e^
3 or more	37%	12%	29%	49%	3.19 (1.97, 5.73)***	1.49 (1.11, 2.08)*	2.15 (1.24, 4.01)*

^a^ Percentage of households with each housing characteristic overall

^b^ Percentage of households within each tenure type with each housing characteristic

^c^ Percentages may not add up to 100 due to rounding

*p < 0.05 **p < 0.01 ***p < 0.001

^d^ Median (Q1, Q3)

^e^ Referent group € adjusted for age, sex, ACCHS, Aboriginality and income

^f^ Binomial category 3+ physical housing problems vs 0–2 problems

^g^ Adjusted Odds Ratio not Prevalence Ratio

^h^ No. of physical dwelling problem domains: structural, damp, electrical, plumbing, no smoke alarm, security, vermin & temperature control

## Results

### Participants

Of the 627 SEARCH carers who completed the carer survey, 600 (96%) provided data on their housing and tenure type and were included in the analyses. There were no statistically significant demographic differences between carers who provided housing data and those who did not. The 600 participants in the current study were the carers of 1406 SEARCH children aged between 0 and 17 at the time of recruitment.

The majority of SEARCH carers were female (91%) (Table 1) and most identified as Aboriginal (78%). Median carer age was 33 years (Q_1_ 27, Q_3_ 39). A total of 30% of carers were employed (full time or part time), 54% performed home duties, 13% were unemployed or unable to work. Most had no formal qualifications (52%).

### Tenure type

The majority of SEARCH carers lived in some form of social housing (60%), with 21% renting privately and 19% in homes owned by a usual member of the household (3.3% owned outright and 16% being paid off) (Table 1). Income was substantially associated with tenure type; those in the lowest income bracket had 7.33 (95%CI 2.95, 18.16) times the odds of living in social housing rather than an owned home, compared to those in the highest income bracket. Similarly, those carers who were not working (OR 3.20, 95%CI 1.16, 8.83) or who performed home duties (OR 3.50, 95%CI 1.85, 6.65) had significantly higher odds of living in social housing than owned homes, compared with those who were employed.

For every additional 10 years of age, carers had significantly lower odds of living in private rent (OR 0.51, 95%CI 0.36, 0.71) or social housing (OR 0.70, 95%CI 0.52, 0.93) compared to an owned home. After adjusting for all other demographic factors, Aboriginal carers had higher odds than non-Aboriginal carers of living in social housing rather than an owned home (OR 4.17, 95%CI 2.25, 7.74) and of living in social housing rather than in a privately rented home (OR 2.79 (95%CI 1.60, 4.86). There was no significant difference in the odds of owning and renting privately for Aboriginal versus non-Aboriginal carers (Table 2). There was some significant variation in tenure type by ACCHS; carers recruited from ACCHS D had over four times the odds of living in social housing rather than an owned home (OR 4.44, 95%CI 2.03, 9.73) when compared with carers from ACCHS A.

### Housing and household characteristics

Tables 2 and 3 show housing characteristics reported by SEARCH carers and the associations between each housing factor and tenure type, after adjustment for age, sex, ACCHS, Aboriginality and income. Most dwellings were houses (94%) rather than apartments (6.5%). Small cell counts prevented statistical analysis of difference by tenure type, though the proportion of apartment-dwellers in private rental was highest at 12%, with apartments accounting for only 1% of owned homes and 7% of social housing homes.

### Mobility

Households renting privately had lived in their homes for fewer years (median 1.1 yrs) than those in social housing (median 3.7 yrs) and owned homes (median 5.0 yrs) (Table 2). Likewise, SEARCH children in private rental at the time of the survey had lived in significantly more homes than those in owned homes or social housing. A SEARCH child living in private rental had 3.31 times the odds (95%CI 2.14, 5.13) of having lived in 4+ houses since birth compare to those living in owned homes. One in eight SEARCH carers reported that they had been forced to move out in the past twelve months; this did not vary significantly by tenure type.

### Affordability

Affordability problems were reported by a high proportion of carers and while income was not significantly associated with housing affordability problems, tenure type was (Table 2). Those in social housing were less likely to report housing affordability problems compared to those in their own homes (PR 0.65 95%CI 0.48, 0.91) and renting privately (PR 0.61, 95%CI 0.46, 0.82).

### Household size, density and crowding

Overall, 30% of carers reported having felt too crowded where they live, with no significant difference by tenure type. Very few carers reported that their home was too big (1.7%, small cell counts prohibited modelling), while 42% of carers reported that their home was too small. Those in social housing were more likely to report that their home was too small than those renting privately (PR1.39, 95%CI 1.06, 1.87).

SEARCH homes had a median of 3 bedrooms and 5 people who normally slept there, with a median household density of 1.3 Persons Per Bedroom (PPB). Measures of household density, PPB and crowding according to the CNOS, did not differ significantly according to tenure type (see Table 2). Overall, 9% of SEARCH households failed the first occupancy standard of the CNOS, having more than two persons per bedroom.

### Physical dwelling conditions

Physical housing problems were most prevalent for those in social housing, who reported a median of 2 problem domains (Q_1_ 1, Q_3_ 4), followed by those in private rental (1 (Q_1_ 0, Q_3_ 3)), while those in owned/mortgaged homes reported the fewest problems (0 (Q_1_ 0, Q_3_ 1) (Table 3). Social housing tenants were more than three times as likely to report 3+ dwelling problem domains than those in their own homes (PR 3.19, 95%CI 1.97, 5.73). Those in private rental were 2.15 (95%CI 1.24, 4.01) times as likely to report 3+ problem domains than those in owned homes.

Vermin was the most commonly reported dwelling problem; 43% of carers reported problems with cockroaches, mice or other vermin. Those in social housing had 4.24 (95%CI 2.39, 7.52) times the odds of reporting a vermin problem than those in their own home (Table 3).

Forty percent of carers reported at least one major structural problem with their dwelling, the most common of which were major cracks in the walls or floors (29%) and sinking or moving foundations (20%). Again, each form of structural problem was significantly more likely to be reported by those in social housing than those in their own home; some were also significantly more likely for those in social housing than those in private rental.

The need for housing to be made more secure was reported by 39% of carers, with those in social housing 3.30 (95%CI 2.03, 5.96) times as likely to report this problem as those in their own home, and 1.67 (95%CI 1.24, 2.36) times as likely as those in private rental.

Damp and mildew was also prevalent in SEARCH households (34%), but disproportionately so for those in social housing (43%) compared to those in their own home (13%, PR 2.90, 95%CI 1.83, 5.00) or private rental (28%, PR 1.53, 95%CI 1.12, 2.18). Those in private rental were also more likely to report damp and mildew than those in owned homes (PR = 1.89, 95%CI 1.11, 3.41).

Over a quarter of carers reported some problem with temperature control (27%). Those in social housing were twice as likely to report being unable to make their homes warm enough in winter (PR 2.05, 95%CI 1.10, 4.33) and 3.95 (95%CI 1.90, 10.10) times as likely to be unable to make their homes cool enough in summer than those in owned homes.

Those in social housing reported the lowest rates of broken or absent smoke alarms (4.3%), the only measure of physical housing function in which social housing was reported to perform better than other tenure types. Both carers in private rental and home ownership were less likely to report having a functioning smoke alarm than those social housing (PR 0.32, 95%CI 0.13, 0.83 and PR 0.37, 95%CI 0.16, 0.84) respectively.

### Associations between other sociodemographic variables and housing factors

Overall, tenure type had more substantial and significant independent associations with housing outcomes than other sociodemographic variables in the models. There were some exceptions, however, the most notable of which was the association between income and home heating, with those in the lowest income bracket 10 times as likely as those in the highest income bracket to report being unable to keep their home warm enough in winter (PR 10.0 95%CI 2.3, 17.6). Those in the lowest income bracket were also 2.5 times as likely to report feeling crowded than those in the highest bracket (PR 2.5 95%CI 1.2, 6.2). Carers with the lowest household income also reported more physical dwelling problems than those in the highest income bracket (PR 1.6 95%CI 1.2, 2.1). Yet income was not significantly associated with housing affordability problems, the ability to keep home cool enough in summer or objective measures of household crowding. Carer age was associated with the number of years lived in current home, although even an additional decade of age (PR 1.5, 95%CI 1.3, 1.6) did not have as substantial an effect as tenure type.

## Discussion

Housing problems were common in this large sample of urban Aboriginal families. The pattern of variance in housing circumstances according to tenure type is striking and paints a clear picture of the pressure points of each tenure type. Home ownership was associated with the fewest physical dwelling problems and the most stable housing, however housing affordability problems were significantly more prevalent than for families in social housing. Conversely, social housing tenure was associated with the lowest prevalence of affordability problems and similar occupancy duration to home ownership, but a significantly higher number of physical dwelling problems. In many respects, those in private rental experienced ‘the worst of both worlds’. Private renters were just as likely as those in their own home to report housing affordability problems yet they reported significantly more physical dwelling problems than those living in their own homes and the most housing instability. These findings generally comport with what is known about the relationships between housing tenure type and other forms of housing need.

Consistent with international literature, home ownership amongst SEARCH participants was associated with life course and socioeconomic factors and increased with both age and income [53]. Carers in social housing were also significantly older than those in private rental, perhaps reflecting the long waiting lists for social housing, which can be over 10 years in many parts of urban NSW [54]. There was no significant difference in income for those in private rental versus social housing, which may reflect the growing and substantial pockets of concentrated poverty in Australia’s private rental market given the contraction of the social housing sector in recent decades [55]. State housing providers recently estimated that they are only able to meet 44% of social housing need in NSW [56]. Indeed in a qualitative study with Aboriginal people in Western Sydney, several families reported paying unaffordable rent in the private market to avoid homelessness while awaiting social housing [31].

The proportion of SEARCH households living in social housing (60%) is slightly higher than in other studies of Aboriginal child health (50%) [36, 57] and more than double that of Aboriginal households in the 2011 census (26%) [35, 36, 57]. However the most stark contrast is observed in relation to non-Aboriginal Australian households, of whom 4% were renting from a social housing provider in the 2011 census [35]. The proportion of SEARCH households in home ownership (19%) is also lower than that of Aboriginal households in the 2011 census (36%) [26].

Around a quarter (25%) of SEARCH children had lived in five or more homes since birth. Similar rates of high residential mobility were found amongst urban families participating in WAACHS (29%) [36]. As has been observed elsewhere [37, 39, 58], housing instability was more common amongst those renting privately than those living in social housing or in their own home. While this is to be expected given the tenuous right to occupancy offered by Australia’s private rental system [55], the implications for SEARCH children in private rental are concerning. Frequent residential moves in childhood are linked with poorer child health, social and emotional wellbeing and educational outcomes; and the long-term negative health and wellbeing effects of childhood residential instability into adulthood have been demonstrated in several large longitudinal studies in Australia and internationally [6, 36, 59–63].

While residential moves can occur when families choose to move to different locales for employment or for positive housing reasons, such as entering home ownership, the experience of lower income families is often characterised by a lack of choice and control [31, 36, 64]. The relatively high proportion of SEARCH families who report being forced to move from where they live in the past 12 months is evidence of unwanted mobility. Housing mobility is often associated with adverse family events such as separation or family violence, though existing research indicates that the main drivers for Indigenous mobility, particularly in urban areas, are housing-related [37]. That is to say, the main reasons families move is to seek more appropriate housing (for instance leaving crowded, unaffordable or poor quality dwellings), being evicted or asked to leave by landlords [37, 65]. This would suggest that housing policy and economic interventions can potentially have a particularly significant impact on the housing instability of urban Aboriginal families.

SEARCH households had more usual residents yet a similar number of bedrooms to the average non-Aboriginal Australian household [35, 66], resulting in higher household density rates. As SEARCH households consist only of families with children, it is perhaps more meaningful to compare them with other households with children; LSIC households had a similar number of usual residents (mean of 5.2), while a representative sample of non-Aboriginal Australian households with children had fewer residents (mean of 4.4) [37]. There are many reasons why Aboriginal households tend to be larger than non-Aboriginal households, including higher birth rates and other social and cultural factors, however housing factors including attempts to cope with high housing costs, poor housing availability and the accommodation of homeless family and friends by have also been documented as drivers for large household size and crowding [31, 66, 67].

The proportion of SEARCH households with more than two persons per bedroom (violating the first standard of the Canadian National Occupancy Standard (9.0%) is almost triple that of the general Australian population (3.4%), though it is lower than that observed in LSIC households (17%) and Aboriginal households in the 2011 census (12.9%) [35, 44]. These differences are likely to be partly due to SEARCH’s urban population, as crowding is known to increase substantially with increased relative isolation [35].

While significant differences in crowding by tenure type have been noted elsewhere [35–37], this was not the case amongst SEARCH participants. This accords with existing evidence that affordability issues in the private housing market can squeeze Aboriginal families into homes too small for their needs [64] and that even households with good incomes and stable housing situations can experience crowding when called on to host family and friends in need [31]. The sizeable discrepancy between the proportion of households who reported having felt too crowded (30%) or that their house was too small (42%) and the relatively low rates of crowding as defined by the CNOS (9%) is a finding of interest. For this and other reasons, crowding measurement is being further scrutinised by the research team elsewhere. The relationship between crowding and health and wellbeing for Aboriginal Australians is complex; while some studies have found mixed or positive associations between crowding and social and emotional wellbeing [27, 66], many others show clear associations between crowding and infectious disease, poor school attendance and performance, family violence and risk of eviction [36, 47, 68].

The high prevalence of housing affordability problems reported here accords with the high cost of housing in Australia, particularly in metropolitan areas. SEARCH carers living in social housing were the least likely to report housing affordability problems. While most low income Australian households in private rental are eligible for Commonwealth Rent Assistance (government contributions towards rental costs) the amounts provided have been described as insufficient to make housing affordable for many low income families [31, 64].

Housing affordability stress has been shown to affect mental health over and above general financial hardship and can also affect health through reduced funds for nutritious food, access to health care and numerous other requirements [69]. There is some evidence that those renting privately in Australia spend the highest proportion of their income on housing costs [39] and experience more psychological distress than home purchasers experiencing the same levels of housing affordability stress [42, 69]. Thus SEARCH households in private rental may experience greater negative impacts due to housing affordability problems than home owners. While this may be due to inherent differences in the characteristics of owners and renters and not necessarily causally related to tenure, the benefits associated with home ownership, including control, stability, prestige and wealth-generation and the relatively weak private rental tenancy protections available in Australia are likely to play a role [42, 64, 66, 69].

Poor physical dwelling conditions have traditionally been the main focus of research into the associations between housing and health [4, 5], particularly in remote Aboriginal communities where significant associations have been found [25]. The prevalence of major structural problems in SEARCH households was notably higher than in the most recent representative survey of Australian housing, the 1999 Australian Housing Survey (AHS) [39]. Major cracks in walls and floors, the most prevalent problem in the AHS, was reported by 7% of respondents, compared to 29% of SEARCH households. Similar discrepancies are noted with other problems including sinking or moving foundations (AHS 5%, SEARCH 20%) and rising damp (AHS 5%, SEARCH 19%). However, the proportions of major structural problems observed in SEARCH were similar to those in the NATSISS, where 25% of Aboriginal people in non-remote areas were living in a dwelling with major structural problems [26], and in the National *Social* Housing Survey, where major cracks in walls or floors were reported by 21% of mainstream public housing tenants and 33% of State Owned and Managed Indigenous Housing (SOMIH). Similarly, rising damp was reported by 20% of those in public housing and 29% of those in SOMIH [70].

These differences may largely be explained by tenure type, as in the current study. Fifty percent of LSIC participants in social housing reported that their homes needed repairs vs 30% of private renters and 19% of those in their own homes [44]. And while WAACHS did not differentiate between those renting privately or from a social housing provider, households that were being rented had 2.5 times the odds of having three or more indicators of poor housing compared to households with a mortgage [43]. Aboriginal participants in a recent study described the social housing in Western Sydney as “generally old and in poor condition” and reported experiencing difficulty obtaining repair and maintenance services [31], which comports with a recent report from the NSW Audit Office about the inadequacy of current funds to maintain current social housing stock [56].

It is perhaps unsurprising that owner-occupied homes tend to be in better condition than other dwellings [66]. This trend may reflect better access to economic resources or the greater incentive home owners have to maintain their dwelling [66]. Incentive and capacity to maintain homes to an adequate standard is perhaps worth considering from a policy stance. The anomaly of smoke alarms being the only physical dwelling condition where social housing performed better than other tenure types may in part be due to the legal requirement for all NSW landlords to install and maintain smoke alarms. This points to the potential power of clear legislation regarding landlord responsibilities in improving housing conditions [71]. It could also be argued that housing standards may play a role. In a recent report of social housing standards, a dwelling could be considered to be of an acceptable standard despite having neither a functioning toilet nor a bath/shower, so long as all other facilities (kitchen sink, laundry tub, fridge and others) were present [70]. We argue that most Australians would not consider a dwelling without such essential facilities to be acceptable.

This study has several limitations. Firstly causal inference cannot be asserted from cross-sectional studies as the direction of influence cannot be proven [25]. Also, as previously indicated, SEARCH families are not a representative sample of urban Aboriginal households in NSW [35]. Thus these findings are best suited for internal comparisons and longitudinal analyses [46, 48]. Recruitment materials did not mention housing problems, so no specific bias is likely with regards to housing outcomes. Study findings are based on self-report survey data, not on the direct or objective observation of homes. There is some evidence to suggest that residents tend to underreport problems with their housing [72] and that new home owners tend to underestimate the cost of repairs and maintenance their homes require [73]. Lastly, the Phase One SEARCH survey did not gather data on the significant issue of family homelessness; one in every 15 Indigenous children in Australia sought homelessness assistance in 2014–2015, most with a parent [74]. That said, SEARCH offers a detailed account of the housing situations of a large sample of urban Aboriginal families and the current study findings have a number of policy implications.

Government decisions affect housing availability, affordability and conditions in myriad ways [75]. Levers available to different levels of government include taxation, tenancy law, zoning law and the provision of various forms of direct housing assistance [38]. Government policies have a particularly large effect on the housing circumstances of Aboriginal Australians, in part because of their overrepresentation as low-income renters either in social housing or as recipients of Commonwealth Rent Assistance [38]. While this means that the aging of social housing stock, reduced funding for social housing maintenance and scaling back of social housing availability [76] has undoubtedly disproportionately affected Aboriginal households, it also means that they may potentially benefit greatly from programs to improve housing affordability, stability and quality, provided they are appropriately targeted and accessible for Aboriginal households [30]. There are many incentives for governments to act to improve housing for its citizens, with evidence that improved housing conditions decrease health and social services use and associated expenditures [77–79].

This study suggests that home ownership for Aboriginal households should be encouraged and assisted where possible and desirable, for example through existing Home Purchase Schemes, such as those available through Indigenous Business Australia or the NSW Aboriginal Housing Office. Nevertheless, some households may still struggle with housing affordability when paying a mortgage and new solutions may be required to support such households [64]. Importantly though, home ownership is not an option for a significant proportion of the urban Aboriginal community [30, 31], as is increasingly the case for a growing proportion of all low and middle-income Australians [55]. Social housing is an important tenure type for urban Aboriginal people, providing much needed, affordable and relatively stable housing. However, a second obvious implication of this study is the need for improved dwelling conditions in social housing [56].

Thirdly, the apparent vulnerability of SEARCH households in private rental to multiple forms of housing disadvantage suggests the need for a variety of policy responses, including increasing the number of social housing dwellings to address high levels of unmet need [56] and encouraging other forms of affordable housing accessible to urban Aboriginal people [80]. Our findings also support growing calls to strengthen Australian tenant protections around affordability, dwelling conditions and secure tenancy, all of which are weak by international standards [55, 81, 82]. Such protections may include rent control, removal of ‘without grounds’ termination by landlords, better protections against racial discrimination and the enforcement of higher dwelling standards [81]. In New Zealand, one means of improving housing conditions in both social housing and private rental is the trial of a rental ‘Warrant of Fitness’, requiring rental properties to pass certain health and safety standards in much the same way that a car must to be allowed on the road [71]. Improved dwelling conditions in social housing and increased social housing supply are both likely to require additional public funds to be invested in social housing. This has not been considered desirable in Australia for many decades, where social housing has a problematic public image [76, 83]. It is possible that as increasing numbers of middle class Australians are priced out of home ownership in metropolitan areas [42], public awareness and support for social and affordable housing, along with the need for improved protections for renters in both the private and social housing sectors, may grow [81, 82].

Lastly, while tenure type is significantly associated with particular forms of housing disadvantage in this study and elsewhere, the fundamental drivers behind Aboriginal housing disparity must also be kept in mind. Aboriginal people are less likely to own their own homes largely due to the ongoing effects of colonisation and dispossession, which includes intergenerational poverty, marginalisation, and ongoing racial discrimination in employment and housing markets [84, 85]. Thus efforts to improve housing disadvantage for Aboriginal people will also likely require cross-sectoral collaboration with the education, employment, justice and health sectors, along with programs to actively address racial discrimination [32, 85].

## Conclusion

The high prevalence of housing problems in this large urban Aboriginal cohort suggest the need for public health, housing and other professionals to join with Aboriginal people and organisations in advocating for improved access to decent and affordable housing. The significant discrepancies in the housing problems reported by households in different tenure types suggest the potential need for differentiated policy responses.

## Additional files

Additional file 1: SEARCH Housing questions for Tenure Type Matters. (PDF 457 kb)

Additional file 2: Analysis Variables for Tenure Type Matters. (PDF 278 kb)

## Abbreviations

ACCHS: Aboriginal Community Controlled Health Service
AH&MRC: Aboriginal Health and Medical Research Council
NSW: New South Wales
SEARCH: Study of Environment on Aboriginal Resilience and Child Health
