# Supplementary material for: Housing conditions of urban households with Aboriginal children in NSW Australia: tenure type matters
Source: BMC Public Health. 2017 Aug 1;18:70. doi: 10.1186/s12889-017-4607-y (PMC5540447; doi:10.1186/s12889-017-4607-y)
Supplement: Supplementary file 1 — SEARCH Housing questions for Tenure Type Matters. (PDF 457 kb) [file 12889_2017_4607_MOESM1_ESM.pdf]

S1. SEARCH Housing questions for Tenure Type Matters

AMS (ACCHS) ☐

Family Number ☐☐☐☐

Study ID Number of Carer ☐☐☐☐☐☐

Interviewer ☐

Date of Interview ☐☐ ☐☐ ☐☐

SEARCH

Study of Environment on Aboriginal Resilience and Child Health

I. Parent/Carer Survey

1a. Age ☐☐ Years

1b. Sex ☐ Male ☐ Female

1c. Are you of Aboriginal or Torres Strait Islander descent?

- ☐ Yes, Aboriginal
- ☐ Yes, Aboriginal and Torres Strait Islander
- ☐ Yes, Torres Strait Islander
- ☐ No

2b. What qualifications do you have? *(cross all that apply)*

- ☐ None
- ☐ Trade/apprenticeship
- ☐ Certificate from college
- ☐ Diploma (beyond Year 12)
- ☐ Bachelor Degree
- ☐ Post Graduate diploma/higher degree
- ☐ Other (please specify) ______________________
- ☐ Refused
- ☐ Don’t know

a. How would you describe your current employment status?

- ☐ Employed full-time (include self employed)
- ☐ Employed part-time (include self employed)
- ☐ Unemployed
- ☐ Student and working
- ☐ Student not working
- ☐ Home duties
- ☐ Retired
- ☐ Unable to work due to health problems
- ☐ Other (please specify) ______________________
- ☐ Refused
- ☐ Don't know

3c. Which of these groupings would best describe your HOUSEHOLD'S income for the past 2 WEEKS from all sources (e.g. wages, CDEP, pensions and study allowances etc)?

Please use prompt card.

- ☐ $1- $199
- ☐ $200 - $399
- ☐ $400 - $599
- ☐ $600 - $799
- ☐ $800 - $1999
- ☐ $2000 and over
- ☐ None
- ☐ Other (please specify) ______________________
- ☐ Refused
- ☐ Don't know

13. Have any of these issues affected you and your family in the past 12 months?

No	Yes	Refused	Don't know	
☐	☐	☐	☐	**k.** You have felt too crowded in where you lived
☐	☐	☐	☐	**p.** You were forced to move out of a place you were living for any reason

15. What best describes your current housing?

- ☐ House
- ☐ Flat, unit, apartment
- ☐ Other (please specify) ______________________
- ☐ Refused
- ☐ Don't know

16. How long have you lived there?

☐☐ Years & ☐☐ Months ☐ Refused ☐ Don't know

17. How many people usually sleep in your current home?

☐☐ People ☐ Refused ☐ Don't know

18. How many bedrooms are in your home?

☐☐ Bedrooms ☐ Refused ☐ Don't know

19. Is your current home:

No	Yes	Refused	Don't know		
☐	☐	☐	☐	**a.**	Owned by you or any usual member of this household?
☐	☐	☐	☐	**b.**	Being paid off by you or any usual member of this household?
☐	☐	☐	☐	**c.**	Rented by you or any usual member of this household?
☐	☐	☐	☐	**d.**	Owned by the Department of Housing?
☐	☐	☐	☐	**e.**	Owned by Aboriginal Housing Office?
☐	☐	☐	☐	**f.**	Owned by Community Housing (Land Council, other Aboriginal housing provider or other community housing provider)?
☐	☐	☐	☐	**g.**	Other (please specify) ______________________

20. Does the home that you live in have any of the following problems?

No	Yes	Refused	Don't know		
☐	☐	☐	☐	**a.**	Too big
☐	☐	☐	☐	**b.**	Too small
☐	☐	☐	☐	**c.**	Needs to be more secure

No	Yes	Refused	Don't know		
☐	☐	☐	☐	**d.**	Rates too expensive
☐	☐	☐	☐	**e.**	Mortgage too expensive
☐	☐	☐	☐	**f.**	Rent too expensive
☐	☐	☐	☐	**g.**	Rising damp
☐	☐	☐	☐	**h**	Damp or mildew on any of the walls, ceilings or windows
☐	☐	☐	☐	**i.**	Major cracks in walls/floors
☐	☐	☐	☐	**j.**	Sinking/moving foundations
☐	☐	☐	☐	**k.**	Sagging floors
☐	☐	☐	☐	**l.**	Walls or windows that are not straight
☐	☐	☐	☐	**m.**	Wood rot/termite damage
☐	☐	☐	☐	**n.**	Major electrical problems
☐	☐	☐	☐	**o.**	Major plumbing problems
☐	☐	☐	☐	**p.**	Cockroaches, mice or other vermin
				**r.**	Other (please specify)__________________________

____

21. Are you able to make your home warm enough in winter?

☐ No ☐ Yes ☐ Refused ☐ Don't know

22. Are you able to make your home cool enough in summer?

☐ No ☐ Yes ☐ Refused ☐ Don't know

23. Is there a functioning smoke alarm installed in your home?

☐ No ☐ Yes ☐ Refused ☐ Don't know

[NB Q4 below is taken from the Child Health Survey completed for participating SEARCH children]

Q 4. Since ___________________ was born how many different houses has he/she lived in?

☐☐ Number ☐ Refused ☐ Don't know
