# Supplementary material for: Housing conditions of urban households with Aboriginal children in NSW Australia: tenure type matters
Source: BMC Public Health. 2017 Aug 1;18:70. doi: 10.1186/s12889-017-4607-y (PMC5540447; doi:10.1186/s12889-017-4607-y)
Supplement: Supplementary file 2 — Analysis Variables for Tenure Type Matters. (PDF 278 kb) [file 12889_2017_4607_MOESM2_ESM.pdf]

Additional File 2. Analysis Variables for Tenure Type Matters

Construct	Variable Name	Survey Question	Response Options	Analysis Categories/ Range
**Carer Demographics**	Age	1a. Age _ _ years	Age in years	[numeric value, discrete number]
	Gender	1b. Sex	Male;<br>Female	0 - Male #<br>1- Female
	Aboriginal and Torres Strait Islander Status	1c. Are you of Aboriginal or Torres Strait Islander descent?	Yes, Aboriginal;<br>Yes, Torres Strait Islander;<br>Yes, Aboriginal & Torres Strait Islander;<br>No	0 - non-Aboriginal# (no)<br>1 - Aboriginal (Yes to any)
	ACCHS	[site noted by research officer]	A; B; C; D	0 - A#, 1 - B, 2 - C, 3 - D
**Socioeconomic Status**	Qualifications	2b. What qualifications do you have?	None;<br>Trade/apprenticeship;<br>Certificate from college;<br>Diploma (beyond Year 12);<br>Bachelor Degree;<br>Post Graduate diploma/higher degree;<br>Other	0 - Bachelor or postgraduate degree#<br>1 - Trade, certificate, diploma<br>2 - None
	Employment Status	3a. How would you describe your current employment status?	Employed full-time (incl self-employed);<br>Employed part-time (incl self-employed);<br>Unemployed;<br>Student and working;<br>Student and not working;<br>Home duties;<br>Retired;<br>Unable to work due to health problems;<br>Other	0 - Employed# (full time or part time)<br>1 - Studying (student working or not working)<br>2 - Home duties<br>3 - Not working (unemployed, retired or unable to work due to health problems)
	Fortnightly income	3c. Which of these groupings would best describe your HOUSEHOLD'S income for the past 2 WEEKS from all sources (e.g. wages, CDEP, pensions and study allowances etc)?	$1-199;<br>$200-399;<br>$400-599;<br>$600-799;<br>$800-1999;<br>$2000 and over;<br>None; Other	0 - $2000+#<br>1 - $800-1000<br>2 - $0-799

Construct	Variable Name	Survey Question	Response Options	Analysis Categories/ Range
**Tenure Type**	Tenure Type	19. Is your current home:	a. Owned by you or any usual member of this household;<br>b. being paid off by you or any usual member of this household;<br>c. rented by you or any usual member of this household;<br>d. owned by the department of housing;<br>e. Owned by Aboriginal Housing Office;<br>f. Owned by Community Housing (Land Council, other Aboriginal housing provider or other community housing provider);<br>g. Other	0 - Owned (a or b)#<br>1 - Private rent (c)#<br>2 - Social housing (d, e, f)
**Dwelling Structure**	Dwelling Structure	15. What best describes your current housing?	House;<br>Flat, unit, apartment;<br>Other	0 - House#<br>1 - Apartment<br>(NB 'House' includes townhouse)
**Mobility**	Duration of residence in current home	16. How long have you lived there?	_ _ Years and _ _ Months	[numeric value – discrete number of months, expressed as years]
	Number of houses child lived in since birth ¥	*Q4. Child Health Survey.*<br>*Since ________ was born, how many different houses has he/she lived in?*	_ _ Number	[numeric value – discrete]<br>0 - 1-3 homes#<br>1 - 4+ homes
	Forced to move in past 12 months	13. Have any of these issues affected you and your family in the past 12 months?<br>You were forced to move out of a place you were living for any reason.	No;<br>Yes	0 - No#<br>1 - Yes
**Affordability**	Affordability problems	Derived variable from<br>20 Does the home that you live in have any of the following problems:<br>d. Rates too expensive<br>e. Mortgage too expensive<br>f. Rent too expensive	No (if no to d, e & f);<br>Yes (if yes to d, e or f)	0 - No#<br>1 - Yes

Construct	Variable Name	Survey Question	Response Options	Analysis Categories/ Range
**Household Occupancy**	Number of usual residents	17. How many people usually sleep in your current home?	_ _ People	[numeric value – discrete]
	Number of Bedrooms	18. How many bedrooms are in your home?	_ _ Bedrooms	[numeric value – discrete]
	Persons Per Bedroom (PPB)	Derived variable from 17 & 18: number of usual residents divided by number of bedrooms	Ratio: people/ bedroom	[continuous ratio]<br><br>0 - 2 or fewer PPB#<br>1 - >2 PPB<br>*(2 or more PPB considered crowded, as per first condition of Canadian National Occupancy Standard (CNOS))*
	Subjective Crowding	13. Have any of these issues affected you and your family in the past 12 months?<br>You have felt crowded in where you lived	No; Yes	0 - No#<br>1 - Yes
	Home too big	20. Does the home that you live in have any of the following problems: a. Too big	No; Yes	0 - No#<br>1 - Yes
	Home too small	20 b. Too small	No; Yes	
**Dwelling Quality**	Major cracks in walls/floors	20 i. Major cracks in walls or floors	No; Yes	0 - No#<br>1 - Yes
	Sinking/moving foundations	20 j. Sinking/moving foundations	No; Yes	0 - No#<br>1 - Yes
	Sagging floors	20 k. Sagging floors	No; Yes	0 - No#<br>1 - Yes
	Walls or windows not straight	20 l. Walls or windows not straight	No; Yes	0 - No#<br>1 - Yes
	Wood rot/termite damage	20 m. Wood rot/termite damage	No; Yes	0 - No#<br>1 - Yes

Construct	Variable Name	Survey Question	Response Options	Analysis Categories/ Range
**Dwelling Quality**	**Structural problems (*Domain)**	Derived variable from Q 20 i - m	No (if no to 20 i-m)<br>Yes (if yes to one or more of 20 i-m)	0 - No#<br>1 - Yes
	Rising damp	20 g. Rising damp		0 - No#<br>1 - Yes
	Damp/mildew on walls, ceilings, windows	20 h. Damp or mildew on any of the walls, ceilings or windows		0 - No#<br>1 - Yes
	**Damp or mildew (*Domain)**	Derived variable from Q 20 g, h	No (if no to 20 g, h)<br>Yes (if yes to one or both of 20 g, h)	0 - No#<br>1 - Yes
	**Major electrical problems (*Domain)**	20 n. Major electrical problems	No; Yes	0 - No#<br>1 - Yes
	**Major plumbing problems (*Domain)**	20 o. Major plumbing problems	No; Yes	0 - No#<br>1 - Yes
	**Vermin (*Domain)**	20 p. Cockroaches, mice or other	No; Yes	0 - No#<br>1 - Yes
	**Needs to me more secure (*Domain)**	20 c. Needs to me more secure	No; Yes	0 - No#<br>1 - Yes
	Unable to make home warm enough in winter	21. Are you able to make your home warm enough in winter?	No; Yes	0 - No<br>1 – Yes#
	Unable to make home cool enough in summer	22. Are you able to make your home cool enough in summer?	No; Yes	0 - No<br>1 – Yes#
	**Temperature control (*Domain)**	Derived variable from 21 & 22	No to either 21 or 22 = yes to temperature control problem	0 - No#<br>1 - Yes
	**No functioning smoke alarm (*Domain)**	23. Is there a functioning smoke alarm installed in your home?	No; Yes	0 - No<br>1 – Yes#
	**Number of Physical Dwelling Problems (of 8 Domains)**	Derived variable – tally of number of physical dwelling problems from each *Domain	Score 1 for every physical dwelling problem *Domain	[numeric value – discrete, score 0-8]0 - 0-2 problems#<br>1 - 3+ problems

referent category *8 Physical Dwelling Problem Domains (structural, damp or mildew, electrical, plumbing, no smoke alarm, security, vermin & temperature control) ¥ note that models including number of houses lived in since birth were also adjusted for child age in months, a variable derived from the child health survey child date of birth question and date of survey.
